# Prediction of mortality after esophagectomy: A comprehensive analysis of various risk scores in a national esophageal center

**DOI:** 10.1016/j.sopen.2025.08.001

**Published:** 2025-08-16

**Authors:** Ahmed Al-Mawsheki, Maximilian Bockhorn, Sorin Miftode, Fadl Alfarawan, Asem Al-Salemi, Catharina Fahrenkorg, Nader- El-Sourani

**Affiliations:** a Department for General and Visceral Surgery, University Hospital Oldenburg, Klinikum Oldenburg AöR, Rahel-Straus-Straße 10, 26133 Oldenburg, Germany.; b Department for General-, Visceral – and Transplantation Surgery, University Hospital Münster, Albert-Schweitzer-Campus 1, 48149 Münster, Germany

**Keywords:** Esophageal cancer, Esophagectomy, Mortality prediction models, O-POSSUM score, Charlson comorbidity index, Risk stratification, Surgical outcomes, Perioperative risk analysis, Prognostic models, Hybrid surgery

## Abstract

**Background:**

Esophagectomy remains the cornerstone treatment for esophageal cancer but is associated with significant perioperative morbidity and mortality, even in specialized centers. Accurate preoperative risk assessment is crucial to improve patient outcomes, and various predictive models are available for risk stratification. This study aimed to validate and compare the performance of nine established predictive models in forecasting 30-day mortality following esophagectomy in a high-volume esophageal cancer center.

**Methods:**

We retrospectively analyzed of 101 patients who underwent esophagectomy between January 2020 and December 2023 was performed. Clinicopathological characteristics and mortality data were obtained. The predictive accuracy of nine risk models, including the Esophageal-POSSUM (O-POSSUM), Charlson Comorbidity Index (Charlson), Postoperative Estimation of Risk (PER), and Fuchs scores, was assessed using logistic regression, Hosmer-Lemeshow tests for calibration, and the area under the receiver operating characteristic curve (AUC) for discrimination. Mann-Whitney *U* tests were used to evaluate significant differences between survivors and non-survivors.

**Results:**

The 30-day mortality rate was 8.91 %. The O-POSSUM and Charlson scores demonstrated the highest predictive accuracy with AUCs of 0.832 and 0.806, respectively. The PER and Fuchs models also showed significant associations with mortality but with moderate predictive ability. Models such as the American Society of Anesthesiologists (ASA) and Philadelphia scores demonstrated limited predictive utility. Significant differences in predictive performance were noted across patient subgroups.

**Conclusions:**

The O-POSSUM and Charlson scores were reliable tools for predicting 30-day mortality after esophagectomy. Other models require further validation and refinement. Tailoring risk assessment models in specific clinical settings may enhance their predictive accuracy and contribute to improved patient outcomes.

## Graphical abstract

**Figure:**
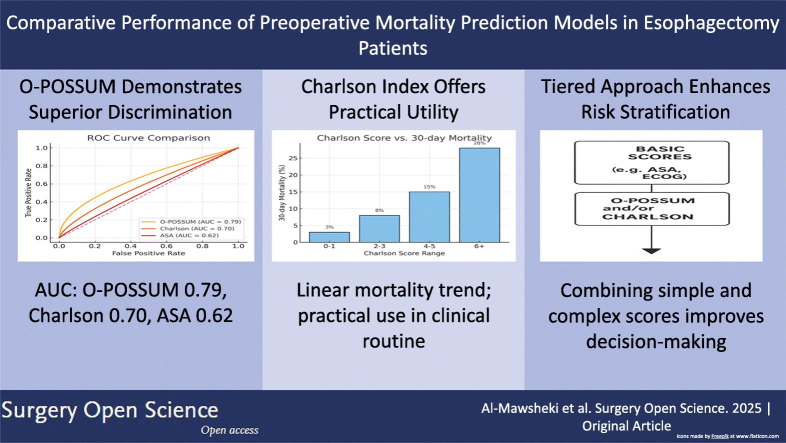

## Introduction

Esophageal cancer is a significant global health concern, with an estimated 604,100 new cases and 544,100 deaths in 2020, according to new estimates From GLOBOCAN 2020 [1]. It ranks among the leading causes of cancer-related deaths and accounts for the sixth highest cancer mortality rate [2], with a five-year survival rate of only 19 %, as reported by the American Cancer Society [3]. Teng et al. (2024) emphasized that population growth and aging substantially increased the burden of esophageal cancer, underscoring the need for effective prevention and control strategies [4]. Esophagectomy remains the cornerstone of multimodal treatment regimens for esophageal cancer. Despite its role as standard care, esophagectomy is associated with significant perioperative morbidity and mortality, even in specialized centers. A systematic review and meta-analysis revealed that a substantial proportion of studies reported postoperative mortality rates greater than 5 %, with some reaching as high as 23.8 % [5].

Significant postoperative complications are reported by the Esophagectomy Complications Consensus Group (ECCG), with a morbidity rate exceeding 40 % and a 90-day mortality rate of 7.6 % [6].

The *2022 National Oesophago-Gastric Cancer Audit* (NOGCA) reported that between April 2018 and March 2021, 3632 esophagectomies were performed, with a 30-day mortality rate of 1.5 % and a 90-day mortality rate of 3.3 % [7]. This is in contrast to an earlier study by Anderson et al. (2018), which reported a higher 90-day mortality rate of 4.2 % [8]. Recent data from NOGCA suggest a reduction in mortality rates over time, reflecting improvements in surgical practice and perioperative care and emphasizing the ongoing efforts to standardize and optimize patient outcomes.

Based on data from the National Cancer Database (NCDB), Keong et al. (2016) explained that hospital volume significantly influences surgical outcomes after esophagectomy. Their analysis revealed that hospitals performing more than 41 esophagectomies annually reported a 30-day mortality rate of 4.3 %, while lower-volume centers reported rates as high as 7.2 % [9]. These data underscore the importance of hospital volume in enhancing postoperative outcomes in patients with esophageal cancer. Recent meta-analyses further support this conclusion, emphasizing the need for the centralization of care to improve survival rates3. Reflecting this, German authorities, referencing data from Deutsches Ärzteblatt, have mandated a minimum annual case load of 26 esophagectomies to enhance the quality of care [10].

Accurate preoperative risk assessment is vital given the high morbidity and mortality rates associated with esophagectomy. Traditional models such as the esophagogastric Physiologic and Operative Severity Score for the enUmeration of Mortality and Morbidity (O-POSSUM score), although widely used, have been criticized for their lack of specificity and predictive accuracy in esophagectomy settings [11]. More specialized models, such as the International Esodata Study Group (IESG) risk prediction model and the Prognostic Risk Evaluation for Esophagectomy (PER) score, integrate diverse variables to offer tailored predictions [12,13]. However, despite its predictive strength, the Rotterdam model relies heavily on clinical and histopathological data, which may limit its applicability to various clinical settings [14]. Additional models, such as the estimation of physiological ability and surgical stress (*E*-PASS score), which include intraoperative parameters such as blood loss, provide comprehensive assessments but may be hindered by their complexity and the extensive data they require [15].

Furthermore, well-established models such as the Charlson Comorbidity Index are pivotal in assessing surgical risk; however, their broad medical focus may not adequately reflect the specialized risks associated with esophageal surgery [16]. The American Society of Anesthesiologists (ASA) score similarly provides a valuable health status classification but may not fully predict postoperative outcomes specific to esophagectomy due to its generalized approach [17].

The Fuchs Risk Score, developed to provide insights into the mortality risk for esophageal cancer patients, offers detailed risk stratification but often demands comprehensive preoperative data, which can limit its utility in less data-rich settings [18]. The Surgical Risk Score (SRS), which is valuable for broad surgical applications, might not address all complications related to esophageal surgeries, given its wider application across various surgical types [19].

Each of these models demonstrates significant strengths in risk stratification, however, their limitations highlight the need to develop more specific tools that address the unique demands of esophagectomy.

The diversity of these models illustrates the challenges in establishing a universally optimal strategy and treatment algorithm. This study aimed to critically analyze and compare several scoring systems to predict 30-day postoperative morbidity and mortality at a national esophageal cancer center and provide a thorough review of the current literature to identify the potential benefits and guide patient selection for esophagectomy. We hypothesized that among the various scoring systems evaluated, some models, specifically those incorporating esophagus-specific risk factors and patient comorbidities, would demonstrate superior predictive accuracy in estimating 30-day morbidity and mortality following esophagectomy compared with more generalized scoring systems. Furthermore, we anticipate that model performance may vary based on individual patient characteristics, such as age, tumor stage, and ASA of Anesthesiologists classification, underscoring the need for tailored risk assessment in esophageal cancer surgery.

## Materials and methods

### Study design and patient population

This retrospective monocentric study analyzed the data of 101 patients who underwent esophageal resection for underlying malignancy between January 2020 and December 2023 at the University Hospital for General and Visceral Surgery, Klinikum Oldenburg, a national esophageal cancer center, were analyzed. Patients who underwent esophagectomy for benign conditions or emergency surgeries were excluded. The study was conducted in accordance with the ethics committee of the University Oldenburg and approved under AZ-2024-086.

### Patient characteristics

Patient data and clinical factors included sex, age, Body Mass Index (BMI), ASA, and Eastern Cooperative Oncology Group performance status (ECOG). Additional factors, such as vital signs, electrocardiogram (ECG) findings, spirometry results, blood analysis, and the presence of comorbidities were recorded. The type of surgery performed, TNM classification after restaging, tumor histology, and neoadjuvant therapy status were also analyzed. Patients with benign conditions were excluded from this study.

### Preoperative workup

Presurgical assessment included physical examination, standard laboratory tests, and detailed presurgical risk stratification. Staging was performed using Upper Endoscopy (UE) with biopsy, Endoscopic Ultrasound (EUS), and computed tomography of the chest and abdomen. All patients presented to the multidisciplinary tumor board, and patients received either a neoadjuvant regime or primary surgery depending on the tumor stage. Neoadjuvant therapy included chemotherapy (CTX), chemoradiotherapy (RCTx), or radiotherapy alone (RT). Most patients receiving CTX were treated with the FLOT protocol, whereas RCTx was based on the CROSS regimen. The choice of therapy was based on tumor location, stage, and MDT recommendation.

### Surgery

Surgery in our tertiary referral center was performed by two experienced surgeons. All patients underwent a curative intended Ivor-Lewis esophagectomy with gastric pull-up including a two-field lymphadenectomy, either open, hybrid, or totally minimally invasive.

### Prediction models

In this study, we evaluated a series of established risk-score models to ascertain their effectiveness in predicting outcomes in patients undergoing esophagectomy. These models included the IESG, PER, Steyerberg (Rotterdam), Philadelphia RA, Fuchs Esophageal Surgery Risk Score, O-POSSUM, and ASA Physical Status Classification. Each of these models offers unique perspectives on patient risk and utilizes various clinical and demographic variables to forecast perioperative outcomes. Importantly, this research aimed to validate the recommendation from the German clinical guidelines regarding the predictive reliability of the O-POSSUM score for esophagectomy.

### Statistical analysis

Nine models are selected and electronically calculated. Mortality rates were also documented. The analysis was performed using the SPSS IBM Statistics 27. The performance of the models was analyzed in terms of calibration and discrimination. Calibration measures how closely the predicted outcomes match actual results. The comparison between the observed and expected (O-E) deaths for each model was analyzed using the Hosmer–Lemeshow (HL) goodness-of-fit test. Higher values of the HL statistic indicate poorer model calibration. In this analysis, a value of *P* < 0.05 was considered to indicate a statistically significant lack of fit. Discrimination refers to the ability to distinguish between patients who will die and those who will survive by applying logistic regression and calculating the area under the receiver operating characteristic (ROC) curve (AUC). Values between 0.7 and 0.8 indicate reasonable or moderate discrimination, and values greater than 0.8 indicate good or excellent discrimination. Additionally, we applied the Mann-Whitney *U* test to assess the discriminative performance of the predictive models, specifically examining whether the scores significantly differed between survivors and non-survivors. This analysis provides insight into the effectiveness of the models in distinguishing between patient outcomes. AI-based tools were used to enhance the grammatical and language clarity of the study.

## Results

### Clinicopathologic characteristics

Table 1 provides a summary of the baseline characteristics of the patient cohort, highlighting a predominance of male patients and a median age of 65 years (range 37–85). Comorbidities were common, particularly cardiovascular disease (68 %) and diabetes (15 %), reflecting the multimorbid profile typical of esophageal cancer patients undergoing surgery.

**Table 1:** Overview of Demographic and Clinical Parameters in Esophagectomy Patients.

Variables, n (%)		n	%	Median (Range)
Age (years)				65 (37–85)
Sex	Male	79	78.22	
	Female	22	21.78	
Risk factors and Comorbidity				
Smoking		37	36.63	
C2-Abuse		10	9.90	
BMI (kg/m^2^)				26 (18–37)
ASA score	2	33	32.67	
	3	62	61.39	
	4	6	5.94	
ECOG	0	44	43.56	
	1	35	34.65	
	2	11	10.89	
	3	11	10.89	
Cardiovascular diseases		69	68.32	
Pulmonary diseases		19	18.8	
Liver disease		7	6.93	
PAVK		11	10.89	
Diabetes		25	24.75	
Type of surgery				
Hybrid		74	73.27	
Open		25	24,75	
Totally minimally invasive		2	1,98	
Malignancy status				
Primary malignancy only		56	55.45	
Primary malignancy + nodal metastasis		40	39.60	
Primary malignancy + distant metastasis		5	4.95	
Pathology				
Adenocarcinoma		88	87.13	
Squamous cell carcinoma		13	12.87	
Neoadjuvant				
No		51	50.5	
Chemotherapy		37	36.63	
Chemoradiotherapy		12	11.88	
Radiotherapy alone		1	0.99	
Urgency				
Elective		98	97.02	
Emergency		3	2.97	

The distribution of ASA and ECOG performance scores shows that a large proportion of patients presented with a significant preoperative risk status, with ASA III being the most frequent classification. The malignancy status ranged from localized disease to regional lymph node involvement and, in some cases, distant metastasis.

Neoadjuvant treatment was administered in the majority of patients; further details are provided in Section 2.3. The surgical approach varied between open, hybrid, and totally minimally invasive esophagectomy, depending on tumor characteristics, patient condition, and surgeon preference.

### Mortality

**Fig. 1:**
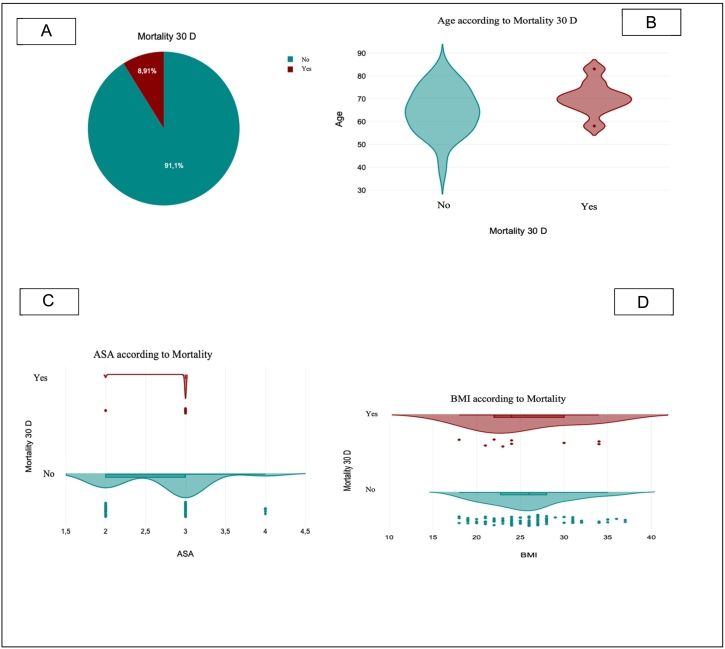
Mortality and their relations to other risk factors. A: Shows 30-day mortality; B: Illustrates the distribution of age according to mortality; C: Demonstrates the ASA score distribution among mortality categories; D: Depicts the distribution of BMI according to mortality.

Postoperative 30-day mortality was observed in 9 patients, corresponding to an overall mortality rate of 8.91 % (Fig. 1A).

Fig. 1 B illustrates the distribution of mortality according to patient age. The highest mortality was observed in the older age groups, particularly among patients aged ≥70 years, while younger patients showed lower mortality rates.

Fig. 1 C shows the distribution of mortality across ASA classifications. Most deaths occurred in patients with ASA III status, which also represented the largest subgroup in the cohort. Fewer deaths were seen in ASA II and ASA IV groups.

Fig. 1 D displays the distribution of mortality according to BMI categories. The majority of fatal outcomes were observed among patients with BMI values in the normal to overweight range (BMI 22–30 kg/m^2^), without a clear clustering in underweight or obese ranges.

Among the three patients who underwent emergency esophagectomy, two died within 30 days. These cases were included in the overall analysis. Regarding tumor histology, 8 out of 9 deaths occurred in patients with adenocarcinoma, reflecting the predominance of this subtype in the cohort.

### Models validation

The comprehensive evaluation of the nine predictive scores for 30-day mortality following esophageal cancer surgery illustrates diverse statistical outcomes and discriminative capabilities. The O-POSSUM score, which accurately predicted a mortality rate of 9 %, was significantly correlated with mortality, with an odds ratio (OR) of 1.19 and an AUC of 0.832. It shows an excellent model fit with a Hosmer-Lemeshow test *p*-value of 0.964. The Rotterdam model also exhibited a positive correlation with mortality (OR = 2.1, AUC = 0.727), backed by a good Hosmer-Lemeshow test result (*p* = 0.798), suggesting that it reliably predicts a higher risk with increasing score values.

The IESG score demonstrated an inverse relationship with mortality (OR = 0.59, AUC = 0.793) with a reasonably good fit (Hosmer-Lemeshow *p* = 0.211), indicating that higher scores may be protective. Similarly, the PER model and Charlson index both displayed positive correlations with mortality (ORs of 2.12 and 1.98, respectively), and both models fit the data well, as evidenced by their Hosmer-Lemeshow *p*-values of 0.451 and 0.903, respectively. The Fuchs score was also significantly associated with mortality (OR = 1.75) and good fit (Hosmer-Lemeshow, *p* = 0.073).

However, the Philadelphia and ASA scores did not achieve statistical significance in their effects, although their models fit adequately with Hosmer-Lemeshow *p*-values of 0.594 and 0.130, respectively. The SRS model did not reach statistical significance, and its fit was moderate (Hosmer-Lemeshow *p* = 0.433). All statistical details, including odds ratios, AUC values, and Hosmer-Lemeshow test results, are shown in Table 2.

**Table 2:** Comparative Overview of Predictive Surgical Risk Models Based on Logistic Regression and Hosmer-Lemeshow Test.

Score	Odds Ratio	AUC	p-Value	Hosmer-Lemeshow p
O-POSSUM	1.19	0.832	0.002	0.964
Rotterdam	2.10	0.727	0.025	0.798
IESG	0.59	0.793	0.002	0.211
PER	2.12	0.777	0.004	0.451
Charlson	1.98	0.806	0.001	0.903
Fuchs	1.75	0.774	0.009	0.073
Philadelphia	1.82	0.606	0.231	0.594
ASA	1.73	0.589	0.384	0.130
SRS	1.86	0.619	0.153	0.433

We further validated the predictive performance of our previously discussed models using the Mann-Whitney U-test, along with effect sizes. Significant differences between survivors and deceased were noted, with the O-POSSUM score illustrating substantial discriminative capacity; the median values were 16 for ‘no’ mortality and 25 for ‘yes’, highlighting its predictive relevance (U = 138.5, *p* = 0.001, *r* = 0.33). Similar significant outcomes were observed in other models, such as Rotterdam, IESG, PER, Charlson, and Fuchs, confirming their efficacy in mortality prediction. However, the Philadelphia, ASA, and SRS models did not show significant differences, indicating a limited predictive utility. The effect sizes ranged from small to medium, reflecting the varied predictive strengths. Detailed results are presented in Table 3.

**Table 3:** Comparative Overview of Predictive Surgical Risk Models Based on Mann-Whitney Test.

Model	U-Value	p-Value	Effect Size r	Outcome (Significance)
O-POSSUM	138.5	0.001	0.33	Significant
Rotterdam	226	0.023	0.23	Significant
IESG	171	0.003	0.29	Significant
PER	142.5	0.005	0.3	Significant
Charlson	159.5	0.002	0.31	Significant
Fuchs	186.5	0.006	0.27	Significant
Philadelphia	323	0.198	0.13	Not significant
ASA	340	0.303	0.1	Not significant
SRS	312	0.163	0.14	Not significant

## Discussion

This study of 101 patients offers insights into the clinicopathologic characteristics, mortality rates, and effectiveness of various models. These findings are contextualized within the existing literature on esophagectomy outcomes and risk stratification, highlighting the need for comparisons with other studies reporting different results.

### Clinicopathologic characteristics

As shown in Table 1, the demographic data revealed a predominantly male cohort (78.2 %), with a median age of 65 years, which is consistent with the literature suggesting a higher prevalence of esophageal cancer in older males. Additionally, research highlights that advanced age (≥75 years) is associated with increased short-term mortality, while sex differences, particularly in squamous cell carcinoma, show that women generally have better prognosis than men [20,21].

The presence of significant comorbidities, such as cardiovascular diseases (68.3 %) and diabetes (24.8 %) (Table 1), highlights the complexity of preoperative management in patients with esophageal cancer. The American Society of Anesthesiologists (ASA) score showed that 61.4 % of the cohort was classified as ASA 3, reflecting the severity of systemic diseases that elevate surgical risks. Although the ASA classification has well-known limitations, such as subjectivity and interobserver variability, it remains widely used in surgical risk assessment due to its simplicity and general applicability. In our study, higher ASA scores correlated with increased 30-day mortality, which supports its relevance as a basic screening tool. However, its prognostic power may be enhanced when combined with other, more objective scoring systems. These findings are consistent with those of previous studies that emphasizes the impact of comorbidities on surgical outcomes. Backemar et al. (2020) underscored the importance of managing comorbid conditions, which are known to affect health-related quality of life (HRQoL) and postoperative outcomes [22]. Respiratory dysfunction is the dominant risk factor for postoperative pulmonary complications [23].

To improve patient outcomes, particularly in those with high ASA classifications, structured prehabilitation programs focusing on nutritional optimization, physical conditioning, and comorbidity control could be implemented in the preoperative phase. Early involvement of multidisciplinary teams may further reduce perioperative risks in vulnerable patients [24].

### Mortality

When comparing our study's findings with those in the existing literature, it is evident that esophagectomy outcomes are influenced by various factors, particularly in high-risk patients. Our study reported an overall 30-day mortality rate of 8.91 % (Fig. 1a), which falls within the range observed in other studies that assessed perioperative outcomes in esophageal cancer surgery, especially considering the influence of hospital and surgeon volume.

Research strongly supports the relationship between higher hospital volume and improved outcomes. For instance, high-volume centers that perform more than 45 esophagectomies annually report significantly lower mortality rates [23]. This aligns with our findings and emphasizes the benefits of centralizing complex surgeries in specialized centers with experienced teams. In contrast, Rodgers et al. (2007) argued that, while hospital volume plays a role, patient-specific factors such as age, comorbidities, and nutritional status are equally, if not more, influential on mortality outcomes [25]. These patient factors highlight the complexity of managing high-risk populations, as seen in our study, with significant comorbidities, such as cardiovascular diseases and diabetes.

Numerous studies have demonstrated that higher hospital volumes in esophagectomy are associated with improved outcomes, including lower postoperative mortality, fewer complications, and better overall management of adverse events [26,27]. Reported volume thresholds vary across the literature, ranging from 10 to over 45 cases annually, yet the trend remains consistent. According to the German S3-guideline, centers performing at least 26 esophagectomies per year are considered high-volume, a criterion met by our institution.

Although the present study is limited to a single-center analysis, this design ensures methodological consistency regarding surgical techniques, perioperative management, and data quality. The uniform institutional environment minimizes variability and enables a focused assessment of different risk scores under standardized clinical conditions.

While this approach precludes direct comparisons with low-volume hospitals, the favorable outcomes observed in our cohort—particularly the low 30-day mortality—are consistent with data reported from other high-volume centers. These findings support the notion that centralized care in experienced units may positively influence short-term surgical outcomes.

Additionally, while most studies support centralization in high-volume centers [28], the role of preoperative risk stratification and personalized patient care is just as critical. For instance, the influence of surgeon expertise was noted to be significant and sometimes even outweighed the volume of the hospital [29].

In our study, emergency esophagectomy (EE) was defined as an unplanned surgical intervention performed under urgent conditions due to acute life-threatening complications. These included esophageal perforation, massive bleeding, or rapidly progressing obstruction with impending perforation. EE was carried out without the standard preoperative optimization typically possible in elective settings.

In our cohort, three patients underwent EE. Of these, two died within 30 days postoperatively, reflecting the significantly higher mortality associated with such procedures. These findings are in line with previous reports, such as Schweigert et al. (2015), which highlight the high risk and complexity of emergency esophageal surgery [30]. As emphasized by Ullah et al. (2023), early detection and multidisciplinary management are essential to improving outcomes in these critical scenarios [31].

### Comparative analysis of predictive models

In a recent systematic review, van Nieuw Amerongen et al. (2024) described more than 33 different risk prediction models developed for estimating morbidity and mortality following esophagectomy. These models vary substantially in structure, clinical scope, and input complexity. While some focus exclusively on preoperative factors, others integrate intraoperative or postoperative elements such as blood loss, contamination, or ICU course. Due to such heterogeneity, not all models are equally suitable for early risk stratification in standard clinical workflows.

In our study, we selected nine risk prediction models based on their relevance in the current literature, clinical applicability, and compatibility with routinely collected data at our center. These included: **O-POSSUM, Rotterdam Score, IESG Score, PER Score, Charlson Comorbidity Index, Fuchs Score, Philadelphia Score, ASA Score, and SRS**. Preference was given to models that rely primarily on preoperative variables, allowing objective comparison across patients without the confounding effects of intra- or postoperative interventions.

Notably, O-POSSUM was included due to its explicit recommendation in the German S3-guideline for the preoperative evaluation of patients undergoing esophagectomy. Some well-known models were excluded because they require laboratory values or intraoperative parameters not systematically documented in our cohort.

A comprehensive comparative overview of the included scoring systems, including phase of assessment (pre/intra/postoperative), data type (objective vs. subjective), and known limitations, is provided in Table 4. This structured summary reflects both the practical and methodological criteria behind model selection in our study and enables transparent contextualization of our findings.

**Table 4:** Comparative Characteristics of Risk Prediction Models Included in This Study.

Score	Assessment Phase	Input Type	Endpoint	Strengths	Limitations
O-POSSUM	Pre/Intra/Postoperative	Objective	30-day Mortality	Well validated, endorsed by German guideline	Complex input, partly intra/post-op
Rotterdam	Preoperative	Objective	30-day Mortality	Simple, preoperative only, widely used in Europe	Limited external validation
IESG	Preoperative	Objective	90-day Mortality	International data, recent development	No intraoperative data, still being validated
PER	Preoperative	Objective	30-day Mortality	Designed specifically for pre-op risk	Lacks intra/post-op insight
Charlson Index	Preoperative	Objective	30-day Mortality	Easy to calculate, broadly validated	No surgical/intra-op data included
Fuchs Score	Pre/Intraoperative	Mixed	In-hospital/30-day Mortality	Tailored to GI surgery, integrates key factors	Limited use outside GI tract, less known
Philadelphia	Preoperative	Objective	30-day Mortality	Developed from esophagectomy-specific data	Limited external validation, rare use
ASA	Preoperative	Subjective	30-day Mortality	Simple and fast clinical estimate	Subjective, interobserver variability
SRS	Preoperative	Objective	30-day Mortality	Surgical-specific risk profiling	Rarely used in practice, not widely validated

Our findings indicated significant variations in the effectiveness of these predictive models. Notably, the O-POSSUM and Charlson scores exhibited the highest predictive accuracies. These findings are consistent with the broad acceptance and application of these models in surgical risk assessment. This is supported by the German National Guidelines on Esophageal Cancer Surgery published in Deutsches Ärzteblatt and foundational research by Charlson et al. [ 10,16] Validation of these models across diverse studies has demonstrated their efficacy and reliability in clinical settings14. In particular, the O-POSSUM score emerged as a particularly strong predictor, with an odds ratio of 1.19 and an area under the curve (AUC) of 0.832, as seen in Table 2, reflecting excellent discrimination. This aligns with findings from other studies that have validated the O-POSSUM score as a reliable predictor of mortality after esophageal cancer surgery [32,33]. While some reviews highlight variability in predictive performance, the O-POSSUM model generally outperforms other risk assessment tools such as the American Society of Anesthesiologists (ASA) and Acute Physiology and Chronic Health Evaluation II (APACHE II), despite occasional over- or under-prediction in specific cohorts [33]. This discrepancy may stem from the failure of these models to account for intraoperative events, which can significantly impact the postoperative outcomes [34]. In contrast, our study's focus on comprehensive preoperative assessments and validation of multiple models may provide a more nuanced understanding of risk stratification.

The Rotterdam model, developed by Steyerberg et al. (2006), is designed for esophageal cancer and integrates patient characteristics and tumor-specific factors to predict surgical outcomes [14]. Despite its detailed focus, our study revealed that the model did not achieve the same predictive accuracy as O-POSSUM. Furthermore, limitations in external validation and application of the model across different patient cohorts have been highlighted [35,36]. Incorporating pathology data, rather than relying solely on administrative data, has been suggested to improve the predictive power [37]. Although the Rotterdam model shows promise for patient stratification, additional refinement is necessary to enhance its clinical utility and ensure better patient outcomes [38,39].

Additionally, the IESG score, designed to predict 90-day postoperative mortality, stratifies patients based on preoperative factors, with mortality rates varying from 1.8 % to 18.2 %10. In our study, we assessed 30-day mortality outcomes and found an inverse correlation between higher IESG scores and mortality (OR = 0.59, AUC = 0.793) (Table 2). This aligns with the model's ability to categorize patient risk, but highlights the challenge of applying a 90-day model to short-term outcomes. Previous systematic reviews, such as Warnell et al. (2015), indicate that many models, including IESG, require further validation and refinement owing to limitations in discrimination and calibration [40]. The importance of the 90-day mortality metric, as noted by Talsma et al. (2014), may explain these discrepancies with other studies. These results suggest that while the IESG model shows potential for patient stratification, its utility for 30-day mortality requires additional validation and context-specific adjustments [41].

In our study, the PER score exhibited strong predictive accuracy for 30-day mortality with an odds ratio of 2.12 and a Hosmer-Lemeshow *p*-value of 0.451, as highlighted in Table 2, confirming its clinical utility. This aligns with previous research by Reeh et al. (2016), who developed the PER score from a cohort of 498 patients, highlighting its significant association with both disease-free survival (DFS) and overall survival (OS) [13]. In our findings, higher PER scores were linked to an elevated risk of postoperative complications, consistent with Reeh's findings, where PER 2 and PER 3 indicated at least double the complication risk compared to PER 1.

However, in emergency cases, such as in patients who are unable to undergo spirometry, the utility of the model may be compromised. This is crucial in our context, where some patients requiring emergency esophagectomy did not have complete preoperative functional evaluations, a limitation also noted in other studies. These findings underscore the need for careful application of the PER score in both elective and emergency settings with room for further refinement to improve its clinical relevance in diverse populations.

Moreover, the Fuchs score showed a positive association with 30-day mortality, with an odds ratio of 1.75 and a Hosmer-Lemeshow *p*-value of 0.073, as shown in Table 2, indicating reasonable predictive ability. This result is consistent with the literature, where the score performed well in high-risk patients, particularly those with comorbidities [18]. However, limitations were observed in healthier cohorts as the score tended to overestimate the risk in these populations. These findings reflect the strengths and weaknesses acknowledged in the original Fuchs study, which also recognized the importance of hospital volume as a crucial preoperative variable. The authors of the Fuchs score argue that this is the first model to incorporate hospital volume as a key factor, providing additional predictive accuracy by including easily available patient characteristics. The simplicity of the score is highlighted as a key advantage, making it useful for preoperative consultation. Despite its ease of use and accuracy, especially in predicting inpatient mortality, limitations such as reliance on administrative data and lack of preoperative nutritional and functional status data have been recognized. These limitations also apply to our findings, where the absence of intraoperative and nutritional data in certain cases (particularly emergency surgeries) may have influenced the accuracy of our predictions.

In our study, the lack of statistical significance for the Philadelphia and ASA scores raised concerns about their predictive reliability in assessing 30-day mortality following esophagectomy. Although the Philadelphia score has shown strong predictive value in Swiss and Australian cohorts [42] and the ASA score has been useful for predicting postoperative complications in transhiatal esophagectomy [43], its predictive power has been inconsistent across other studies [44]; [45]. These discrepancies suggest the need for further validation and refinement of both models in different surgical settings to enhance their clinical applicability.

The application of the Mann-Whitney *U* test to validate the predictive performance of various models, as shown in Table 3, provides a rigorous and reliable statistical approach to this analysis. Specifically, it revealed significant differences between survivors and non-survivors, with particular emphasis on the O-POSSUM score, underscoring its predictive value. As a non-parametric alternative to the *t*-test, the Mann-Whitney U test is well suited for comparing independent groups, such as patient outcomes in this case [46]. Beyond a simple comparison, its adaptability to clustered data and the validation of models in pattern recognition enhances its relevance [47]; [48]. This comprehensive comparative analysis highlighted the variability in the performance of these models, emphasizing the necessity for context-specific validation. The nuanced strengths and limitations of each model, when evaluated alongside the existing literature, fuel ongoing discussions on refining predictive tools for diverse surgical populations.

### Variations in model performance and future research directions

The variability observed in the predictive model performance can be partially attributed to the homogeneous study cohort and the limited sample size. As highlighted in several studies, small datasets often lead to statistical issues, such as overfitting, where models perform well on the training set but fail to generalize to new data [49]. These problems are compounded by biased predictor selection and unstable model specifications, which ultimately result in poor validation performance when applied across different populations [50]; [51]. In our analysis, models such as Philadelphia, ASA, and SRS were underperformed, possibly because of their inability to adapt to the specific clinical characteristics of our patient cohort.

Nevertheless, the O-POSSUM and Charlson scores demonstrated robust predictive capabilities, highlighting their clinical utility in stratifying patients according to their risk levels. This aligns with the findings of multicenter validation studies, which have underscored the benefits of such models in refining surgical decision-making [45]. For future applications, integrating established models, such as O-POSSUM and Charlson, with advanced biomarkers and genetic profiling holds promise for further improving predictive accuracy. By employing larger sample sizes, more sophisticated statistical techniques, such as cross-validation, and careful selection of predictor variables, predictive models can become more reliable and applicable across diverse clinical settings [47]; [52].

### Study strength und limitations

This study features a robust analytical framework that enhances the precision and applicability of perioperative risk assessments across multiple established models, utilizing advanced statistical tools such as the Mann-Whitney *U* test for methodologically rigorous results. Conducted at a high-volume national esophageal cancer center, it leverages a rich dataset for the nuanced evaluation of model effectiveness in real-world settings. However, its monocentric design may limit generalizability to other environments with diverse patient demographics, and its retrospective nature introduces potential biases from historical data that could skew the evaluations. Additionally, the exclusion of models requiring unavailable data narrows the research scope, potentially missing valuable insights into risk stratification, while the complexity of statistical techniques may hinder interpretability and practical application without specialized expertise.

Based on our findings, we recommend the use of both the O-POSSUM and Charlson Comorbidity Index as part of a standard preoperative risk assessment in patients undergoing esophagectomy. These tools demonstrated reliable predictive performance for 30-day postoperative mortality and may assist in shared decision-making, multidisciplinary planning, and perioperative optimization.

Among the evaluated models, O-POSSUM showed the most consistent predictive accuracy, aligning with its current recommendation in the German S3-guideline. Although widely used due to simplicity, scores such as ASA and ECOG are limited by subjectivity and interobserver variability, and should be considered only as initial screening tools.

For practical implementation, we propose a tiered approach: basic scores (e.g., ASA, ECOG) may be used for rapid initial evaluation, followed by application of O-POSSUM and/or Charlson Index in patients with complex comorbidities or elevated perioperative risk.

Integration of these scores into electronic health records (EHRs) could support automated risk stratification, facilitate structured preoperative pathways, and enhance multidisciplinary decision-making. Future multicenter studies are warranted to externally validate our findings and investigate the impact of score-guided management on both short- and long-term outcomes.

## Conclusions

This study enhances the understanding of predictive models for esophageal cancer surgery. The O-POSSUM and Charlson scores demonstrated a high discriminatory power and generalizability across various clinical settings, making them essential for preoperative risk stratification. However, further research and validation are required to assess their clinical utility of other promising models. The variability in model performance underscores the influence of local protocols and patient demographics, emphasizing the need for context-specific adjustments and multi-center studies to refine these tools for better patient outcomes in surgical oncology.

## Abbreviations

AUC: Area Under the Curve
ASA: American Society of Anesthesiologists
BMI: Body Mass Index
ECG: Electrocardiogram
ECOG: Eastern Cooperative Oncology Group
ECCG: Esophagectomy Complications Consensus Group
EUS: Endoscopic Ultrasound
GLOBOCAN: Global Cancer Observatory
HL: Hosmer and Lemeshow
HRQoL: Health-Related Quality of Life
IESG: International Esodata Study Group
MIC: Minimally Invasive Surgery
NCDB: National Cancer Database
NOGCA: National Oesophago-Gastric Cancer Audit
O-E: Observed and Expected
O-POSSUM: Esophagogastric Physiologic and Operative Severity Score for the Enumeration of Mortality and Morbidity
PAVK: Peripheral Arterial Vascular Disease
PER: Prognostic Risk Evaluation for Esophagectomy
ROC: Receiver Operating Characteristic
SRS: Surgical Risk Score
UE: Upper Endoscopy

## CRediT authorship contribution statement

**Ahmed Al-Mawsheki:** Writing – review & editing, Writing – original draft, Software, Resources, Methodology, Investigation, Data curation, Conceptualization. **Maximilian Bockhorn:** Validation. **Sorin Miftode:** Validation, Supervision, Conceptualization. **Fadl Alfarawan:** Supervision, Investigation. **Asem Al-Salemi:** Software, Methodology, Formal analysis, Data curation. **Catharina Fahrenkorg:** Resources, Formal analysis. **Nader- El-Sourani:** Writing – review & editing, Writing – original draft, Validation, Resources, Project administration, Investigation, Conceptualization.

## Informed consent statement

Informed Consent Statement: Informed consent was obtained from all subjects involved in the study.

## Institutional review board statement

The study was conducted in accordance with the Declaration of Helsinki, and approved by the Institutional Review Board of the University Hospital for General and Visceral Surgery Klinikum Oldenburg (protocol code AZ-2024-086, approved on 28 May 2024.).

## Author contributions

**Conceptualization:** Ahmed Al-Mawsheki, Nader El-Sourani - Development of the research question and theoretical framework.

**Methodology:** Ahmed Al-Mawsheki - Designing and planning the methods for data collection and analysis.

**Software:** Asem Al-Salemi, Ahmed Al-Mawsheki - Development and customization of software tools needed for analysis.

**Validation:** Nader El-Sourani, Sorin Miftode, Maximilian Bockhorn - Verification of data and methods for accuracy and reliability.

**Formal Analysis:** Ahmed Al-Mawsheki, Asem Al-Salemi - Performing statistical analysis and data interpretation.

**Investigation:** Ahmed Al-Mawsheki - Collection and evaluation of experimental data.

**Resources:** Ahmed Al-Mawsheki - Procurement of financial and material resources.

**Data Curation:** Asem Al-Salemi - Management and preparation of research data.

**Writing – Original Draft Preparation:** Ahmed Al-Mawsheki - Drafting the initial manuscript.

**Writing – Review & Editing:** Ahmed Al-Mawsheki, Nader El-Sourani - Critical revision and enhancement of the manuscript for important intellectual content.

**Supervision:** Fadl Alfarawan, Maximilian Bockhorn, Nader El-Sourani - Oversight of the research project and mentorship support.

**Project Administration:** Ahmed Al-Mawsheki - Management and coordination of the research project.

## Declaration of Generative AI and AI-assisted technologies in the writing process

During the preparation of this work, the author used ChatGPT-4o to improve spelling, grammar, sentence structure, and to minimize typographical errors. After using this tool, the author carefully reviewed and edited the content as necessary and assume full responsibility for the final content of the publication.

## Funding

This research received no external funding.

## Declaration of competing interest

The authors declare no conflicts of interest.

## Data Availability

The data supporting the reported results are not publicly available due to privacy and ethical restrictions in accordance with data protection policies.
